# HIF1α controls steroidogenesis under acute hypoxic stress

**DOI:** 10.1186/s12964-025-02080-8

**Published:** 2025-02-13

**Authors:** Stephen Ariyeloye, Deepika Watts, Mangesh T. Jaykar, Cagdas Ermis, Anja Krüger, Denise Kaden, Barbara K. Stepien, Vasileia Ismini Alexaki, Mirko Peitzsch, Nicole Bechmann, Peter Mirtschink, Ali El-Armouche, Ben Wielockx

**Affiliations:** 1 Institute of Clinical Chemistry and Laboratory Medicine, University Carl Gustav Carus and Medical Faculty, Technische Universität Dresden, Fetscherstrasse 74, Dresden, 01307 Germany; 2 Department of Pharmacology and Toxicology, Faculty of Medicine, Technische Universität Dresden, Dresden, 01307 Germany; 3 Experimental Centre, Faculty of Medicine, Technische Universität Dresden, Dresden, 01307 Germany

**Keywords:** Hypoxia-inducible factors, MicroRNA, Oxygen sensors, Adrenal gland, Steroidogenesis

## Abstract

**Background:**

Hypoxia is a critical physiological and pathological condition known to influence various cellular processes, including steroidogenesis. While previous studies, including our own, have highlighted the regulatory effects of Hypoxia-Inducible Factor 1α (HIF1α) on steroid production, the specific molecular mechanisms remain poorly understood. This study investigates the role of hypoxia and HIF1α in steroid biosynthesis across multiple experimental models during acute exposure to low oxygen levels.

**Methods:**

To assess the extent to which acute hypoxia modulates steroidogenesis, we employed several approaches, including the Y1 adrenocortical cell line, and a conditional HIF1α-deficient mouse line in the adrenal cortex. We focused on various regulatory patterns that may critically suppress steroidogenesis.

**Results:**

In Y1 cells, hypoxia upregulated specific microRNAs in a HIF1α-dependent manner, resulting in the suppression of mRNA levels of critical steroidogenic enzymes and a subsequent reduction in steroid hormone production. The hypoxia/HIF1α-dependent induction of these microRNAs and the consequent modulation of steroid production were confirmed in vivo. Notably, using our adrenocortical-specific HIF1α-deficient mouse line, we demonstrated that the increase in miRNA expression in vivo is also directly HIF1α-dependent, while the regulation of steroidogenic enzymes (e.g., StAR and Cyp11a1) and steroid production occurs at the level of protein translation, revealing an unexpected layer of control under hypoxic/HIF1 α conditions in vivo.

**Conclusions:**

These findings elucidate the molecular mechanisms underlying acute hypoxia/HIF1α-induced changes in steroid biosynthesis and may also be useful in developing new strategies for various steroid hormone pathologies.

**Supplementary Information:**

The online version contains supplementary material available at 10.1186/s12964-025-02080-8.

## Background

The hypoxia signaling pathway is essential for cellular and systemic adaptations to reduced oxygen levels and for the modulation of many pathophysiological processes [1]. Regulation of this critical system is primarily orchestrated by the activities of the hypoxia-inducible factor (HIFα) subunits, which are in turn tightly regulated by the HIF prolyl hydroxylase domain proteins (PHDs), the von Hippel-Lindau tumor suppressor protein (VHL), and the factor inhibiting HIF1 (FIH) under physiological oxygen concentrations, leading to HIFα inactivation and rapid degradation [2, 3]. However, under reduced oxygen levels, the functionality of these regulators is hampered, which activates HIFα signaling [4]. One of the pivotal processes regulated by the hypoxia/HIF pathway is steroidogenesis in which steroid hormones are synthesized by the catalytic conversion of cholesterol [5]. Steroid hormones play a critical role in controlling several important developmental and physiological processes and any disruption in their production can lead to various health problems, including reproductive disorders, obesity, and hypertension [6].

We, along with others, have previously described a significant role for HIFs in the production of steroids [7–11]; specifically, in a recent study, we revealed a critical function for HIF1α in regulating the synthesis of virtually all steroids in the mouse adrenal gland under steady-state conditions. Additionally, a lack of HIF1α in the adrenocortical cells resulted in a sustained increase in the expression of steroidogenic enzymes, which in turn boosted steroid production. Conversely, HIF1α stabilization in these cells markedly decreased steroidogenesis, as evidenced by a significant reduction in the transcription of the enzymes involved in steroid catalysis and a consequent decrease in steroid output. While available literature also indicates that the hypoxia/HIF pathway can hinder steroid production, the precise molecular mechanisms by which this pathway affects steroidogenesis in acute situations are not well understood [12].

MicroRNAs (miRNAs/miRs) are a class of non-coding ribonucleic acids (ncRNAs) that are recognized for their role in the negative regulation of gene expression [13]. These molecules function primarily by binding to complementary sequences within the 3' untranslated regions (3’UTR) of their target messenger RNAs (mRNAs), resulting in the repression of gene expression either by interfering with translation or by facilitating the degradation of the target mRNA [14]. MicroRNAs are pivotal in controlling a wide array of biological processes, such as angiogenesis, apoptosis, hematopoiesis, and growth and differentiation of cells [12, 15, 16]. Furthermore, alterations in miRNA activity have been linked to various diseases including cancer, diabetes, metabolic and cardiovascular disorders [17]. Under hypoxic conditions, the expression of a specific subset of microRNAs, known as hypoxia-induced microRNAs or hypoxaMIRs, is modulated [18, 19]. These hypoxia-regulated microRNAs are critical for modulating cellular responses to low oxygen levels and ensuring proper adaptation [20]. Additionally, miRNAs are known to play a regulatory role in steroid production, with several studies highlighting their impact on the regulation of genes involved in steroidogenesis [21, 22]. Thus, while available literature indicates that a complex interplay between hypoxia/HIFs and miRNAs influences steroidogenesis [23, 24], a complete understanding of this crosstalk in the regulation of steroidogenesis is still lacking.

Hence, we aimed to elucidate the molecular pathways by which hypoxia adversely affects steroidogenesis by exposing steroidogenic models to acute hypoxic conditions. Results from an adrenocortical cell line model demonstrate that hypoxia critically suppresses steroidogenesis in a HIF1α-dependent manner, which is associated with the direct upregulation of a class of miRNAs targeting a set of key enzymes involved in steroid production. In vivo mouse models confirmed that this increase in miRNA expression under acute hypoxic conditions is directly reliant on HIF1α. Confirming studies in conditional HIF1α-deficient mice revealed that modulation of this process during the acute phase of hypoxic stress is primarily mediated by a HIF1α-dependent regulation of StAR and Cyp11a1 translation, revealing an elaborate layer of regulatory complexity.

## Methods

### Cell culture

Murine Y1 adrenocortical cells were cultured in Dulbecco´s Modified Eagle`s Medium (DMEM) supplemented with 10% Fetal Calf Serum (FCS), 2.5% horse serum, and 1% penicillin–streptomycin, at 37 °C in a humidified incubator with 5% CO_2._ DMEM was additionally supplemented with 2.5% UltroSerG (15,950–017, Pall Life Sciences) and 1% Insulin-Transferrin-Selenium during experiments. For hypoxia experiments, cells were exposed to 5% O_2_ levels in a 37 °C incubator with 5% CO_2_ (Whitley H35 Hypoxystation) (Don Whitley Scientific Limited, United Kingdom). Dimethyloxaloylglycine (DMOG, Biomol) was used at 1 mM.

### Lentivirus production and transduction

Lentiviral particles were generated using HEK-293 T cells (obtained from the American Type Culture Collection, ATCC). To transfect HEK-293 T cells for lentivirus production, a Polyethylenimines (PEI)-mediated transfection protocol was followed. A total of 24 µg plasmid DNA was mixed in 2.4 mL serum-free DMEM. The DNA mixture included 6.5 µg of pMD2.G, 7.5 µg of psPAX2 (both from Addgene) that served as packaging plasmids and 10 µg of pLKO.1 construct bearing the shRNA against HIF1α (Sigma, clone TRCN0000232222) or a non-functional scrambled sequence as a control (ShScr). Separately, 40 µL of PEI stock solution (1 mg/mL, prepared from PEI powder, Sigma, Cat. #408,727) was prepared. The PEI solution was added to the DNA mixture, and the solution was briefly vortexed to ensure thorough mixing. The DNA-PEI mixture was incubated at room temperature for 10 min to allow the formation of DNA-PEI complexes.

During the incubation period, the medium in the HEK-293 T culture dish was replaced with 6 mL of DMEM supplemented with 0.5% fetal calf serum (FCS) to support the cells during the transfection process. After the incubation of the DNA-PEI mixture, the solution was added dropwise to the cells. The culture dish was gently swirled to ensure even distribution of the DNA-PEI complexes across the cell monolayer. The cells were then returned to the incubator and maintained at 37 °C with 5% CO_2_ for 4–6 h, after which the medium was replaced with complete medium including 10% FBS and 1% Penicillin–Streptomycin. Protein expression was assessed 72–96 h post-transfection to confirm successful shRNA delivery and expression. Y1 cells were infected with medium containing lentivirus carrying either shHIF1α or shScr at a 1:1 dilution and incubated overnight. The medium was then removed, and the cells were washed 5 times with fresh medium and incubated under hypoxia (5% O_2_) or normoxia for 24 h, after which the cells were harvested for subsequent analysis.

### Mice

Mice used in this study were bred and housed under specific pathogen-free (SPF) conditions at the Experimental Centre of the Medical Theoretical Center (MTZ, Technical University of Dresden-University Hospital Carl-Gustav Carus, Dresden, Germany. The Akr1b7:cre-*Phd2/Hif1* ^*ff*^/^*ff*^ (P2H1^Ad.Cortex^) mouse line was generated by crossing Akr1b7:cre mice to *Phd2* ^*f/f*^ and *Hif1α* ^*f/f*^ as described elsewhere [7]. All mice (both genders—including WT) used in this study were bred on a C57BL/6 J background (backcrossed at least 10 times) and pups were born at normal Mendelian ratios. The primers used to genotype transgenic mice have been described previously [7]. The isolated adrenal glands were snap frozen in liquid nitrogen and stored at − 80 °C for gene expression or hormone analysis. Breeding of the mice and animal experiments were in accordance with local guidelines on animal welfare and were approved by the Landesdirektion Sachsen, Germany.

### Hypoxia treatment of mice

Wild-type mice were challenged with 9% O_2_ for 24 h, and adrenal glands were collected, snap-frozen, and stored at − 80 °C. To achieve hypoxia (9% O_2_), mice were placed in a BioSpherix chamber. Oxygen pressure within the chamber was consistently sensed using a ProOx P110 compact oxygen controller (Parish, New York) and adjusted according to the set point by nitrogen infusion. Mice were placed in the chamber in standard cages containing bedding and supplied with food and water ad libitum.

### Steroid hormone measurement

Steroids hormones in cell culture supernatants, mouse plasma and adrenal glands were measured as previously described [25, 26].

### Western blotting

For Western blot, adrenal tissue samples were prepared as previously described [26] and protein concentrations were measured using the bicinchoninic acid assay (BCA) (Thermo Fisher). Briefly, 20 µg of protein were separated on a 4–12% Bis–Tris protein gels (Thermo Fisher) and transferred. The membranes were blocked (5% milk) and incubated with primary antibodies against StAR (~ 28 kDa, 1:1000 – Cat. #8449) and Cyp11a1 (~ 55 kDa, 1:1000 – Cat. #14,217) or Metavinculin (~ 145 kDa, 1:1000 – Cat. #18,799) (all Cell Signaling), HIF1α (~ 110–125 kDa, 1:1000 – Cat. #10,006,421) (Cayman chemical), HIF2α (~ 118 kDa, 1:800 – Cat. # NB100-122) (Novus Biologicals), α-tubulin (~ 55 kDa, 1:4000 – Cat. # T5168-100UL) (Sigma Aldrich) overnight at 4 °C. The following day, membranes were washed and incubated with secondary rabbit IgG HRP (R&D Systems) or mouse IgG HRP (Cell Signaling) for 1 h at room temperature. The membranes were then exposed to Fusion Fx (peqlab, VWR). Quantification was performed using Fiji (ImageJ distribution 1.52 K) [27].

### RNA extraction and qPCRs

RNA was isolated from adrenal glands or Y1 cells using the RNA Easy Plus micro kit (Qiagen) (Cat. # 74,034, Qiagen), the RNA Easy Plus mini kit (Cat. # 74,134, Qiagen), the PARIS™ Kit (Thermo Fisher Scientific) or Universal DNA/RNA/Protein Purification kit (cat no. E3597) from EURx. Reverse transcription was carried out with the iScript cDNA Synthesis Kit (BIO-RAD, Feldkirchen, Germany). Gene expression analysis was performed by quantitative PCR with the ‘Ssofast Evagreen Supermix’ (BIO-RAD, Feldkirchen, Germany). Real-time qPCR Detection System-CFX384 (BIO-RAD, Feldkirchen, Germany) was used for quantification of synthesized cDNA. All mRNA expression levels were calculated relatively to beta-2 microglobulin (*B2m*) or eukaryotic translation elongation factor 2 (*Eef2*) housekeeping genes using the 2(-ddCt) method, where ddCT was calculated by subtracting the average control dCT (e.g., 21% O_2_ for Y1 cells, WT or P2H1^Ad.Cortex^), from dCT of all samples individually. Primer sequences used are described in supplementary material (Supplementary Table 1).

### MicroRNA qPCR

RNA was isolated from adrenal glands or Y1 cells using the RNA Easy Plus micro kit (Cat. # 74,034, Qiagen), the RNA Easy Plus mini kit (Cat. # 74,134, Qiagen) or the PARIS™ Kit (Thermo Fisher Scientific). Reverse transcription was carried out using the Mir-X™ miRNA First Strand Synthesis Kit (Takara Bio). The TB Green kit (Takara Bio) was used for detecting miRNA expression levels**.** Relative gene expression was determined using the 2(− ddCt) method with U6 as the housekeeping gene. Primer sequences used are described in supplementary material (Supplementary Table 1).

### Bioinformatic analysis

The miRDB (www.mirdb.org) and TargetScan (www.targetscan.org) computational algorithms were used to identify predicted targets of microRNAs. MicroRNA sequences were determined using the miRPathDB 2.0 (www.mpd.bioinf.uni-sb.de), Genescript (www.genscript.com) and MirGeneDB (www.mirgenedb.org) databases, as well as from published reports [28–35].

### miRNA mimics

Y1 cells were transfected with 30 pmol miRNA-212-3p mimics, miRNA-6924-5p mimics or miRNA mimic negative control (Ctrl) (all Thermo Fisher) for 24 h using the Lipofectamine® RNAiMAX reagent (Thermo Fisher) according to the manufacturer's instructions. Cells were harvested for subsequent qPCR analysis of *StAR, Cyp11a1* and *Cyp21a2*. As predicted by our in-silico analysis, miRNA-212-3p mimics significantly reduced the expression of *StAR* in Y1 cells, whereas miRNA-6924-5p reduced *Cyp11a1*. In addition, the latter also significantly reduced *Cyp21a1* expression (Supplementary Fig. 1 A-B).

### Statistical analyses

All data are presented as mean ± SEM. For assessing the statistical significance of two experimental groups, a two-tailed Mann–Whitney U-test or unpaired t-test with Welch’s correction (after testing for normality with the F-test) was used, unless stated otherwise in the text. For assessing the statistical significance of multiple experimental groups with one variable, the 1-way ANOVA t-test followed by the Tukey’s post hoc test was used. Statistical differences presented in the figures were considered significant at *p*-values below 0.05. All statistical analyses were performed using the GraphPad Prism v10.02 for Windows or higher (GraphPad Software, La Jolla California USA, www.graphpad.com).

## Results

### Hypoxia induces miRNA expression and represses steroidogenesis in Y1 cells

**Fig. 1:**
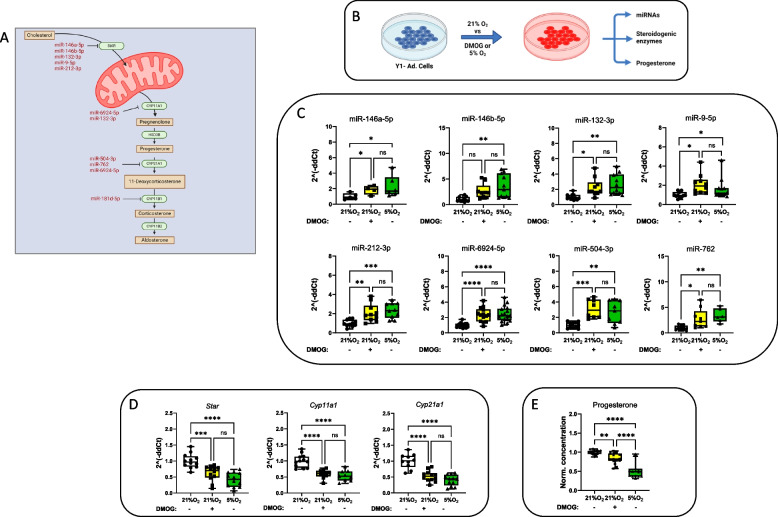
Hypoxia induces microRNA expression and represses steroidogenesis in Y1 cells. **A** Schematic representation of the identified microRNAs putatively repressing steroidogenic enzymes; miR-146a-5p, miR-146b-5p, miR-132-3p, miR-9-5p, and miR-212-3p target *star*, miR-6924-5p targets *cyp11A1*, miR-504-3p and miR-762 target *cyp21a1*, miR-181d-5p targets c*yp11b1*. **B** Schematic representation of the experimental set-up. **C** qPCR analysis showing a general upregulation of the expression of the identified microRNAs in Y1 cells cultured under hypoxic conditions (5% O_2_ or DMOG) compared to the normoxic group. **D** qPCR-based mRNA expression analysis of key steroidogenic enzymes in Y1 cells cultured under hypoxic conditions (5% O_2_ or DMOG) compared to the normoxic group. **E** Reduction in progesterone secretion in Y1 cells exposed to hypoxic conditions (5% O_2_ or DMOG) compared to the normoxic group. Values are expressed as mean ± SEM (*p < 0.05, **p < 0.01, ***p < 0.001, ****p < 0.0001). One-way ANOVA with the post hoc Tukey’s test. *P* values of miR-146a-5p and miR-9-5p were calculated using a one-tailed Mann–Whitney U-test. All data were normalized to the normoxic group (21% O_2_) average value

Several studies have documented the impact of hypoxia on steroid production across various steroidogenic models [7–9]. Yet, the precise molecular pathways through which hypoxia suppress steroidogenic genes remain incompletely understood, although prior research suggests that HIF1 may primarily play an indirect role [36, 37]. Given the significant role of miRNAs as epigenetic regulators that can negatively influence gene expression, and the proposed interaction between HIFs and miRNAs in mediating gene suppression [5], we aimed to explore the effects of hypoxia on miRNA expression; specifically those targeting steroidogenic enzymes. Through bioinformatic analysis, literature review, and in-house testing (detailed in the Methods section), we identified several microRNAs predicted to target steroidogenic enzymes. Among these, we selected five microRNAs that target *StAR* mRNA due to its pivotal role in catalyzing the rate-limiting step of steroid production [38]. Previous studies have also highlighted the effects of some of these microRNAs on the expression of steroid biosynthetic enzymes [21, 39, 40]. Figure 1A provides an overview of the identified microRNAs, their associated steroidogenic enzyme targets, and corresponding interactions.

Next, to ascertain responsiveness to hypoxia, we evaluated the transcriptional activity of these miRNAs in Y1 cells under hypoxic conditions by exposing them to low oxygen (5% O_2_) or treating them with the hypoxia mimetic DMOG for 24 h compared to the cells from normoxic (21% O_2_) conditions. (Fig. 1B). DMOG stabilizes HIFs even in the presence of high oxygen levels due to blocking PHD enzyme activity thereby mimicking the molecular effects of hypoxia. Compared to normoxic control samples, selected miRNAs showed an overall upregulation in the expression, both under hypoxia and with DMOG (Fig. 1C). Note: We did not test for miR181d-5p at this stage, as Y1 cells do not express *Cyp11b1*. To further investigate the impact of these hypoxia-induced microRNAs on their target steroidogenic enzymes, we measured mRNA levels of the key enzymes in Y1 cells and found a dramatic reduction in all mRNA levels measured (Fig. 1D). Notably, this reduction was associated with a decrease in progesterone, the only measurable steroid in this setting. (Fig. 1E). Taken together, these observations support the notion that hypoxia plays a critical role in suppressing steroidogenesis by enhancing miRNA expression.

### Hypoxia-modulated microRNA expression and steroidogenesis is mediated by HIF1α

**Fig. 2:**
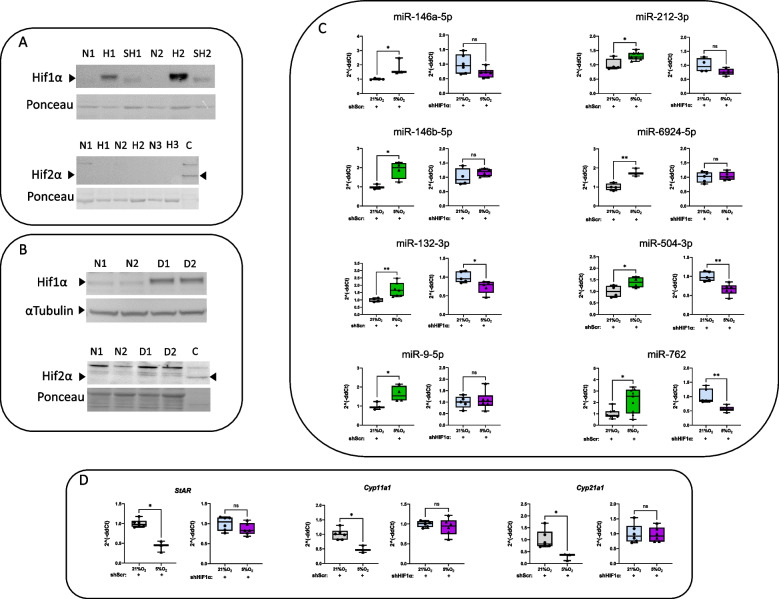
Hypoxia-modulated microRNA expression and steroidogenesis is mediated by HIF1α. **A** Representative Western blot images showing HIF1α but not HIF2α induction in Y1 cells treated with low oxygen pressure (5% O_2_) or **B** DMOG, compared to normoxia treated cells. (*N* = normoxia, H = 5% O_2_, SH = shHIF1α-treated Y1 cells at 5% O_2,_ D = DMOG, C = positive control). **C** qPCR analyses showing an expected upregulation of all 8 miRNAs in shScr-treated Y1 cells after 24 h at 5% O_2_ compared to normoxia. Conversely, this over-expression was lost in shHIF1α-treated Y1 cells exposed to 5% O_2_ compared to the same cells under normoxic conditions. **D** qPCR analysis showing an expected reduction in the expression of S*tAR, Cyp21a1,* and *Cyp11a1* in shScr-treated Y1 cells after 24 h at 5% O_2_ compared to normoxia. There were no significant differences in the expression of the steroidogenic enzymes in shHIF1α-treated Y1 cells exposed to 5% O_2_ compared to the same cells under normoxic conditions. Values are expressed as the mean ± SEM (*p < 0.05, **p < 0.01). All data were normalized to the representative normoxic group (21% O_2_)

To determine the relative contribution of HIFα to the regulation of these miRNAs and steroidogenesis in Y1 cells under acute hypoxic stress, we first determined the expression levels of HIF1α and HIF2α in cells treated with low oxygen pressure (5% O_2_) or DMOG and compared them with their normoxic counterparts using Western blot analysis. While hypoxia or DMOG treatment shows a clear increase in HIF1α levels, HIF2α expression was virtually undetectable in Y1 cells under all conditions (Figs. 2A-B and Supplementary Fig. 2A-D). Based on these findings, we decided to silence the HIF1α expression in Y1 cells using lentiviruses targeting HIF1α mRNA (shHIF1α) (Fig. 2A and Supplementary Fig. 2A) in order to define the impact of this transcription factor on the expression of the miRNAs and the available steroidogenic enzymes. Interestingly, the induction of miRNA expression observed in control cells (shScr) after hypoxia (5% O_2_ for 24 h) (Fig. 2C) (in agreement with the data shown in Fig. 1C) was completely lost in hypoxic Y1 cells treated with shHIF1α (Fig. 2C). Furthermore, while the expression of *StAR*, *Cyp11a1* and *Cyp21a1* was significantly increased in shScr cells (Fig. 2D) (in line with the data presented in Fig. 1D), the expression of these enzymes remained unchanged in Y1 cells in which HIF1α was silenced (Fig. 2D). Taken together, our data clearly indicate that the enhanced expression of a set of miRNAs during acute hypoxic stress, which is associated with reduced steroidogenesis in Y1 cells, is directly mediated by HIF1α.

### Diverse responses to hypoxia-induced miRNA expression and steroidogenesis in vivo

**Fig. 3:**
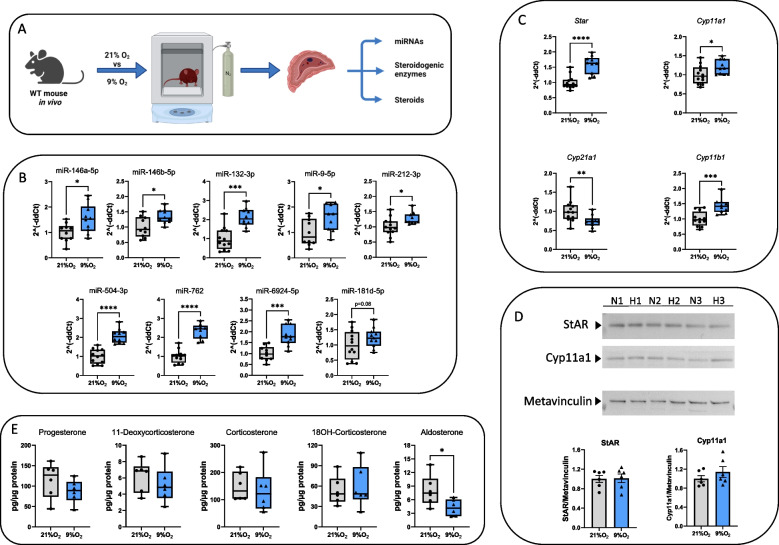
Acute hypoxia regulated steroidogenesis in WT mice in vivo. **A** Schematic representation of the experimental set-up. **B** qPCR results demonstrating a uniform increase in the expression levels of the identified microRNAs (except for miR-181d-5p) in the adrenal glands of mice exposed to low oxygen pressures (9% O_2_) for 24 h compared to the normoxic group. **C** qPCR analysis of the steroidogenic enzyme expression in the adrenal glands of mice exposed to low oxygen pressures (9% O_2_) for 24 h compared to the normoxic group. **D** StAR and Cyp11A1 protein levels were analyzed via Western blot using Metavinculin as a loading control. All protein levels were quantified using FIJI and normalized to Metavinculin levels. *N* = 21% O_2_, H = 9% O_2_ (representative blot with 6 individual samples). **E** Steroid levels in the adrenal glands of mice exposed to hypoxic stress (9% O_2_) for 24 h did not experience a corresponding increase in the first 24 h of exposure compared to the normoxic group (*n* = 6). Values are represented as the mean ± SEM (*p < 0.05, **p < 0.01, ***p < 0.001, ****p < 0.0001). *P* values of were calculated using a Mann–Whitney U-test or one-tailed unpaired t-test with Welch’s correction (miR-181d-5p). All data in B, C and D were normalized to the normoxic group (21% O_2_) average value

We next aimed to explore the dynamics of miRNA expression and activity, and its effects on steroidogenesis under a more complex systemic condition. Therefore, we expanded our investigation to an in vivo setting by acutely exposing WT mice to a reduced oxygen environment (9% O_2_) for 24 h. Readouts from these mice were compared with those from littermate controls maintained at atmospheric conditions (Fig. 3A). First, we examined the expression profiles of HIF1α and HIF2α in adrenal extracts and show that, in parallel with the results obtained in Y1 cells, Western blot analysis demonstrates a clear induction of HIF1α but not of HIF2α after hypoxia treatment (Supplementary Fig. 3A-C), suggesting a primary HIF1α effect.

Analysis of blood cell content in circulation revealed that this acute exposure to hypoxia was too short to substantially increase red blood cell (RBC) formation although it reduced myeloid cell numbers and enhanced platelets (Supplementary Fig. 4A). In line with our cell culture work, in vivo studies revealed a virtually uniform increase in the levels of miRNAs and confirming their sensitivity to acute hypoxic stress (Fig. 3B). Intriguingly, despite broad induction of miRNAs, their impact on the mRNA expression of their corresponding steroidogenic enzymes presented a complex pattern as mRNA levels of 3 out of 4 key enzymes were significantly increased, contrasting with the general expectation of miRNA-mediated suppression (Fig. 3C). Therefore, we performed western blot analyses for StAR and Cyp11a1 enzymes and found that, despite significant increases in mRNA levels, their protein levels remained unchanged (Fig. 3D and Supplementary Fig. 4B), suggesting hypoxia/HIF1-dependent interference during the translation process. In accordance with these results, steroid levels in the adrenal glands remained largely unchanged, and notably, aldosterone levels were significantly diminished, highlighting the intricate regulatory effects induced by acute hypoxia in a living mouse (Fig. 3E).

### In vivo HIF1α mediates miRNA expression independent from steroidogenesis during acute hypoxia

**Fig. 4:**
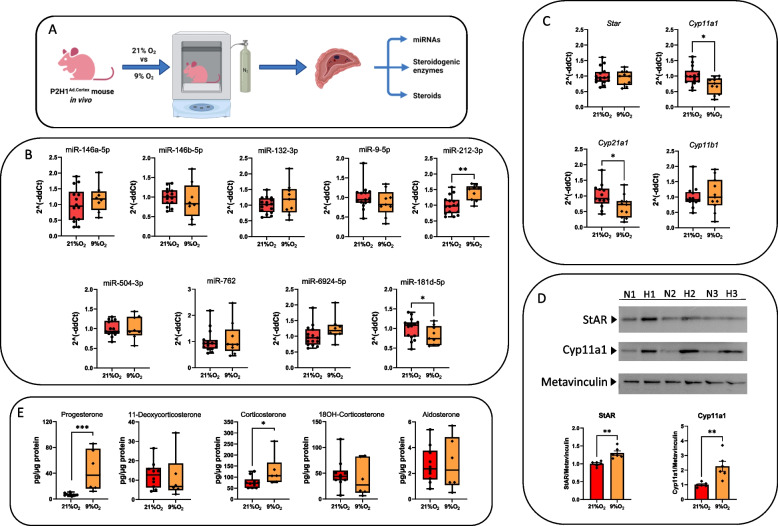
Acute hypoxic modulation of microRNA/steroidogenesis in vivo is regulated in a HIF-1α dependent manner. **A** Schematic representation of the experimental setup. **B** qPCR analysis shows 8 out of the 9 identified microRNAs displaying no changes in their expression levels under both normoxic and hypoxic conditions in P2H1^Ad.Cortex^ adrenal glands after acute hypoxia (9% O2) (24 h) compared to their normoxic counterparts. **C** qPCR analysis of the expression levels of S*tAR, Cyp21a1, Cyp11A1* and *Cyp11b1* steroidogenic enzymes in P2H1^Ad.Cortex^ adrenal glands after acute hypoxia (9% O_2_ for 24 h) compared to their normoxic counterparts. **D** StAR and Cyp11A1 protein levels were analyzed via Western blot using Metavinculin as a loading control. All protein levels were quantified using FIJI and normalized to Metavinculin levels. *N* = 21% O_2_, H = 9% O_2_ (representative blot with 6 individual samples). **E.** Steroid production in the P2H1^Ad.Cortex^ adrenal glands after acute hypoxia (24 h) compared to their normoxic counterparts. Values are represented as the mean ± SEM (*p < 0.05, **p < 0.01, ***p < 0.001, ****p < 0.0001) Mann–Whitney U-test or one-tailed unpaired t-test with Welch’s correction (miR-181d-5p). All data in B, C and D were normalized to the normoxic group (21% O_2_) average value

Building on the expression profile of HIF1α and HIF2α in Y1 cells and WT mice following acute hypoxia, we utilized our previously described mouse model in which HIF1α is specifically deficient in adrenocortical cells, while HIF2α activity is elevated even under normoxic conditions (Akr1b7:cre-*Phd2/Hif1* ^*ff*^/^*ff*^ (P2H1^Ad.Cortex^)) [7]. These P2H1^Ad.Cortex^ mice were subjected to acute hypoxia (9% O_2_ for 24 h) and compared with mice of the same genotype under atmospheric conditions (Fig. 4A). Firstly, we showed that the blood cell pattern in these mice was similar to that in the experiment with WT mice (Supplementary Fig. 5A). However, hypoxic exposure of these P2H1^Ad.Cortex^ mice displayed no changes in expression levels of the microRNAs compared to atmospheric conditions, indicating a direct influence of HIF1α in adrenocortical cells on microRNA expression (Fig. 4B). Consistently, the mRNA levels of *StAR* and *Cyp11b1* enzymes remained largely unchanged under hypoxic conditions. However, we found a significant decrease in the expression of *Cyp11a1* and *Cyp21a1* (Fig. 4C). Contrasting with these mRNA patterns, protein levels of StAR and Cyp11a1 were significantly increased compared to their normoxic counterparts (Fig. 4D and Supplementary Fig. 5B), aligning with the observed increases in progesterone and corticosterone levels in the adrenal glands after acute hypoxic exposure (Fig. 4E).

**Fig. 5:**
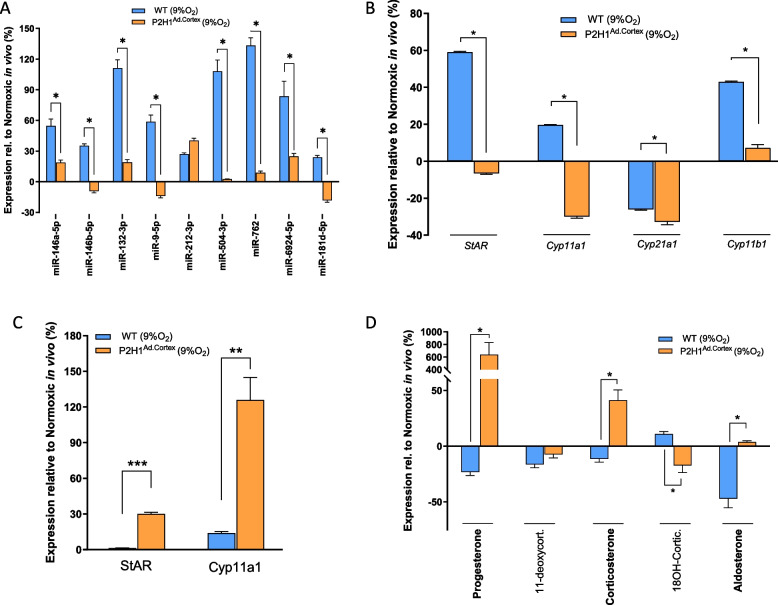
Acute hypoxic and HIF1α regulation of steroidogenesis in vivo. **A** qPCR analysis of microRNAs from adrenal glands of hypoxia treated WT and P2H1^Ad.Cortex^ mice (24 h at 9% O_2_) compared to their respective normoxic counterparts (*n* = 10–16). **B** Gene expression analysis of key steroidogenesis enzymes from adrenal glands of hypoxia treated WT and P2H1^Ad.Cortex^ mice (24 h at 9% O_2_) compared to their respective normoxic counterparts (*n* = 10–16). **C** Protein expression analysis of StAR and Cyp11a1 from adrenal glands of hypoxia treated WT and P2H1^Ad.Cortex^ mice (24 h at 9% O_2_) compared to their respective normoxic counterparts (*n* = 6). **D** Steroids produced from adrenal glands of hypoxia treated WT and P2H1 mice (24 h at 9% O2) compared to their respective normoxic counterparts (*n* = 6–10). Values are represented as the mean ± SEM (*p < 0.05, **p < 0.01) Mann–Whitney U-test

These in vivo experiments prompted further detailed evaluations between the WT and P2H1^Ad.Cortex^ results in order to enhance our understanding of the effects of acute hypoxia and HIF1α on steroidogenesis. When we compared the expression patterns of all microRNAs at 24 h after hypoxia, we observed that, except for miR-212-3p, the expression of all other miRNAs was directly associated with the presence of HIF1α (Fig. 5A). In addition, we found that the mRNA levels of four key enzymes involved in steroidogenesis were also dependent on HIF1α, although their expression patterns contrasted with those of the microRNAs (Fig. 5B). Remarkably, and in contrast to their mRNA levels, both StAR and Cyp11a1 proteins were significantly increased in the cKO mice (Fig. 5C), leading to significantly higher end product steroids—progesterone, glucocorticoid and aldosterone (Fig. 5D). Taken together, these data suggest that HIF1α is a limiting factor in the ultimate translation of these steroidogenic enzymes, and establish HIF1α as a critical, versatile regulator at multiple stages of steroidogenesis during the first 24 h of acute hypoxia in vivo.

## Discussion

In this study, we explored the acute hypoxic response and its potential repressive effects on steroidogenesis across an in vitro and an in vivo model. Our research demonstrates, for the first time, that the induction of a specific cluster of microRNAs is dependent on HIF1α, which correlates with decreased steroidogenic enzymes in Y1 adrenocortical cells. Furthermore, using WT and P2H1^Ad.Cortex^ mice, we identified an additional HIF1α-dependent translational wregulation of StAR and Cyp11a1. Collectively, our findings reveal novel insights into how HIF1α regulates steroidogenesis during the acute phase of hypoxia across various cellular, organ, and systemic contexts.

MicroRNAs are known to be potent post-transcriptional regulators of gene expression, and they primarily act by silencing specific target genes by enhancing their degradation [41] or inhibiting their translation [42]. Several studies using different model systems have investigated the activities of hypoxia-inducible miRNAs [43, 44], and the role of microRNAs in the modulation of steroidogenesis has been documented [45, 46]. Although recent research suggests that hypoxia/HIF may influence steroidogenesis by modulating miRNA expression, comprehensive studies providing a mechanistic understanding of steroidogenesis at the cellular, tissue, and systemic levels have not been performed.

Our study revealed that in response to acute hypoxia, a distinct set of miRNAs targeting at least four steroidogenic enzymes are upregulated across the different settings. This upregulation is primarily controlled by HIF1α, introducing a novel aspect to our understanding of how hypoxia signaling can intervene very early in the steroidogenesis process. This aligns with existing literature on hypoxia-responsive microRNAs, miR-212 and miR-132, which were previously described to regulate steroidogenesis [21, 39]. However, a number of microRNAs used in this study have not yet been implicated in the control of steroidogenesis [47–49]. The induction of these miRNAs in our experimental settings is believed to lead to the repression of steroid biosynthesizing enzymes, thereby reducing steroid production —a hypothesis that was confirmed in Y1 cells under acute hypoxia. Hypoxia/HIF1α-dependent suppression of gene expression is largely, if not entirely, indirect [36, 37], and our results are consistent with previous studies in steroid-producing human H295R cell lines and the marine medaka ovary [23, 24, 50], emphasizing the indirect impairment of steroid production by hypoxia/HIF1α and miRNAs.

Interestingly, we noted a multifaceted response between hypoxia/HIF1α-induced miRNA expression, enzyme expression patterns and steroidogenesis in vivo, highlighting the complex nature of steroidogenesis under acute hypoxic conditions in a complex system. Contrary to the direct correlation between miRNA upregulation and decreased steroid production observed in Y1 cells, our findings suggest the involvement of additional HIF1α-dependent regulatory layers in vivo. This observation was evident as 24-h hypoxia resulted in significantly enhanced mRNA levels of the *StAR* and *Cyp11a1* enzymes but not at the protein level. However, in the absence of HIF1α in cortical cells we found increased enzyme expression opposite to their mRNA concentration. Importantly, the relationship between specific HIF isoforms and miRNAs is highly context dependent. Factors such as cell type, organismal differences, oxygen concentrations, and the duration of hypoxic exposure contribute to the variability [51].

At this stage, it is reasonable to speculate that, in response to acute hypoxia in the mouse, steroidogenesis is regulated by an additional HIF1α-associated regulatory system. For instance, it has been described that mammalian target of rapamycin complex 1 (mTORC1), a central mRNA-to-protein translation activator, is inhibited in hypoxia and affects the global translation rate of mRNAs [52]. It has also been demonstrated that global mRNA-to-protein translation elongation rates can be decreased due to eEF2 inhibition under hypoxic conditions [53]. In addition, miRNAs can also bind to the 3' untranslated regions (UTRs) of their target mRNAs and repress their translation. This interaction can block translation initiation or elongation, leading to reduced protein synthesis while maintaining high mRNA levels [42]. To the best of our knowledge, while hypoxia-mediated post-translational repression has been proposed in other contexts, it has not yet been described in relation to steroidogenic markers such as StAR or Cyp11a1. More research is therefore warranted to better understand which regulatory mechanisms are at play in the hypoxia-HIF1α-miRNA-steroidogenesis axis, especially in complex biological systems such as a living animal.

Taken together, our data clearly demonstrates that HIF1α negatively regulates steroid production during the acute phase of hypoxia in an adrenocortical cell line, and the adrenal gland of mice. How hypoxia/HIF1α specifically affects these different processes during steroidogenesis, and whether the novel set of steroidogenic miRNAs identified in this current work has additional regulatory effects, as has been suggested for mir-132-3p, remains to be unraveled [21]. This complex regulatory web highlights the need for further research to elucidate the specific interactions between hypoxia/HIF1α, microRNA dynamics, and steroidogenic pathways that shape the physiological response to low oxygen levels in a nuanced and context-dependent manner.

## Conclusion

Our study demonstrates that HIF1α plays a critical role in modulating steroidogenesis under acute hypoxic conditions, primarily through the induction of specific microRNAs and their regulation of key steroidogenic enzymes. This reveals an unexpected layer of control, offering new insights into the molecular mechanisms of hypoxia-induced changes in steroid biosynthesis.

## Supplementary Information

Supplementary Material 1

Supplementary Material 2

## Data Availability

Data is provided within the manuscript or supplementary information files. Materials are available upon reasonable request (Ben.Wielockx@tu-dresden.de)

## Abbreviations

ANOVA: Analysis of variance
*B2m*: Beta-2 microglobulin
BCA: Bicinchoninic acid assay
cDNA: Complementary DNA
CO2: Carbon dioxide
CYP11A1: Cytochrome p450 family 11
CYP11B1: Steroid 11β-Hydroxylases
CYP11B2: Aldosterone synthase
Cyp21a1: Cytochrome p450, family 21, subfamily a, polypeptide 1
Eef2: Eukaryotic translation elongation factor 2
FCS: Fetal calf serum
FIH: Factor inhibiting hif1
FIJI: Imagej
HIF: Hypoxia-inducible factor
HRP: Horseradish peroxidase
kDa: Kilodalton
KO: Knock out
MIR/miRNAs: Microribonucleic acid
mTORC1: Mammalian target of rapamycin complex 1
MTZ: Medical theoretical center
PCR: Polymerase chain reaction
PHD: Prolyl hydroxylase domain enzyme
RBC: Red blood cell
RNA: Ribonucleic acid
SEM: Standard error of the mean
SPF: Specific pathogen-free
STAR: Steroidogenic acute regulatory protein
UTR: Untranslated regions
VHL: Von hippel-lindau
WT: Wild type
