# Supplementary material for: HIF1α controls steroidogenesis under acute hypoxic stress
Source: Cell Commun Signal. 2025 Feb 13;23:86. doi: 10.1186/s12964-025-02080-8 (PMC11827267; doi:10.1186/s12964-025-02080-8)
Supplement: Supplementary file 1 — Supplementary Material 1 [file 12964_2025_2080_MOESM1_ESM.docx]

**Supplementary Table 1**

Online Resource II: Primers for mRNA qPCR analysis

| **Primer name** | **Primer sequence (5’ – 3’)** |
| --- | --- |
| StAR_Fwd  StAR_Rev | TCGCTACGTTCAAGCTGTGT  GCTTCCAGTTGAGAACCAAGC |
| Cyp11a1_Fwd  Cyp11a1_ Rev | GCTGAGTACTGGAAAGGGAGC TGCCCAGCTTCTCCCTGTAA |
| mCyp21a1_Fwd  mCyp21a1_Rev | AACAGAACCATTGAGGAGGCCTTGA  TCTCCAAAAGTGAGGCAGGAGATGA |
| Cyp11b1_Fwd  Cyp11b1_Rev | CAGATTGTGTTTGTGACGTTGC  CGGTTGAAGTACCATTCTGGC |
|  |  |

Online Resource II: Primers for miRNA qPCR analysis

| **Primer name** | **Primer sequence (5’ – 3’)** |
| --- | --- |
| mmu-miR-146b-5p | GCTGAGAACTGAATTCCATAGGCT |
| mmu-miR-146a-5p | TGAGAACTGAATTCCATGGGTT |
| mmu-miR-212-3p | TAACAGTCTCCAGTCACGGCCA |
| mmu-miR-6924-5p | ACACTCCAGCTGGGAGAGGATGGGGATTTGG |
| mmu-miR-504-3p | GGGAGAGCAGGGCAG |
| mmu-miR-762 | GGGGCTGGGGCCGGGACAGAGC |
| mmu-miR-181d-5p | GCAAACATTCATTGTTGTCGGT |
| mmu-miR-132-3p | TAACAGTCTACAGCCATGGTCG |
| mmu-miR-9-5p | TCTTTGGTTATCTAGCTGTATGA |
|  |  |
