# Supplementary material for: HIF1α controls steroidogenesis under acute hypoxic stress
Source: Cell Commun Signal. 2025 Feb 13;23:86. doi: 10.1186/s12964-025-02080-8 (PMC11827267; doi:10.1186/s12964-025-02080-8)
Supplement: Supplementary file 2 — Supplementary Material 2 [file 12964_2025_2080_MOESM2_ESM.pptx]

## Slide 1
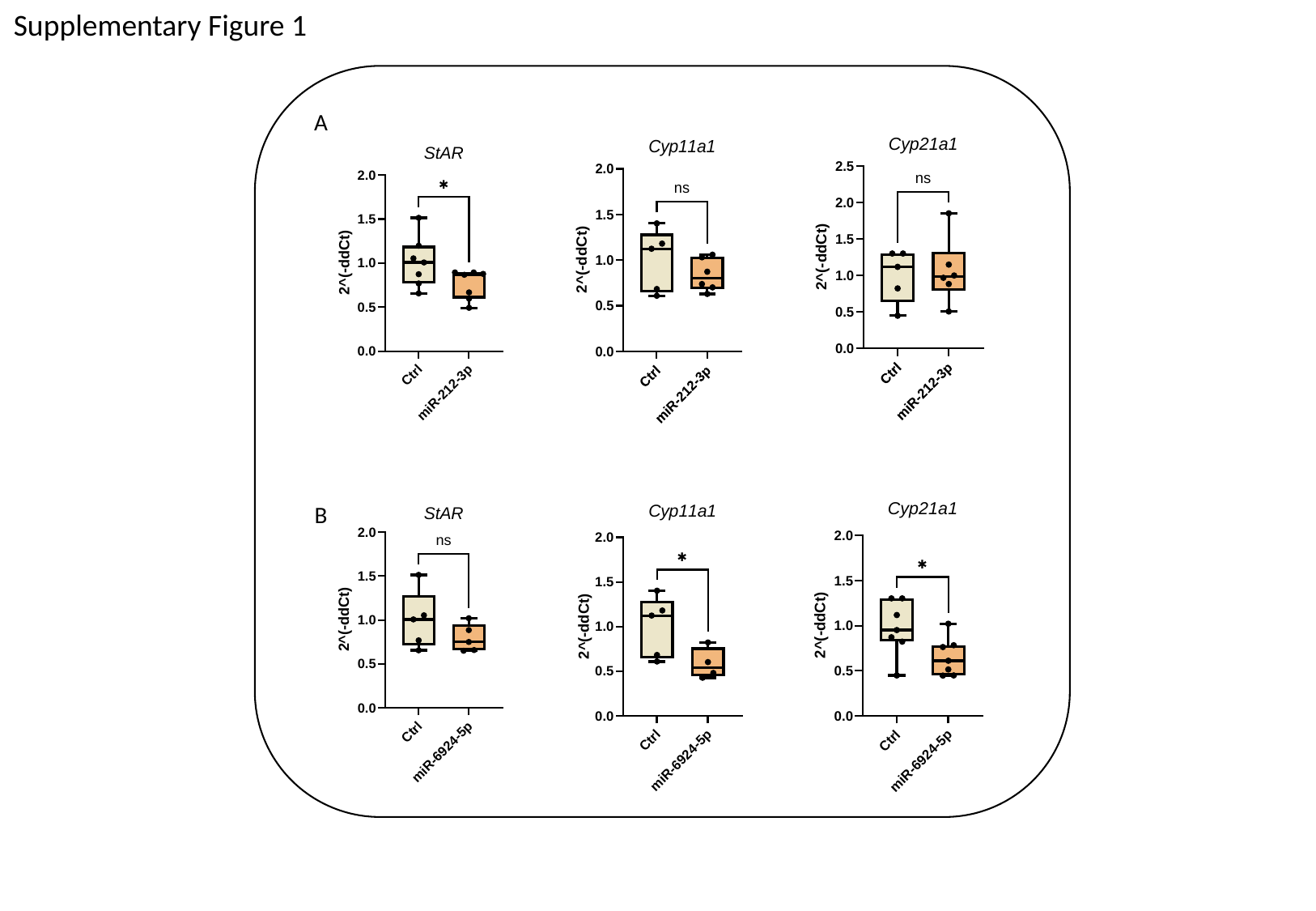

Supplementary Figure 1
A
B

## Slide 2
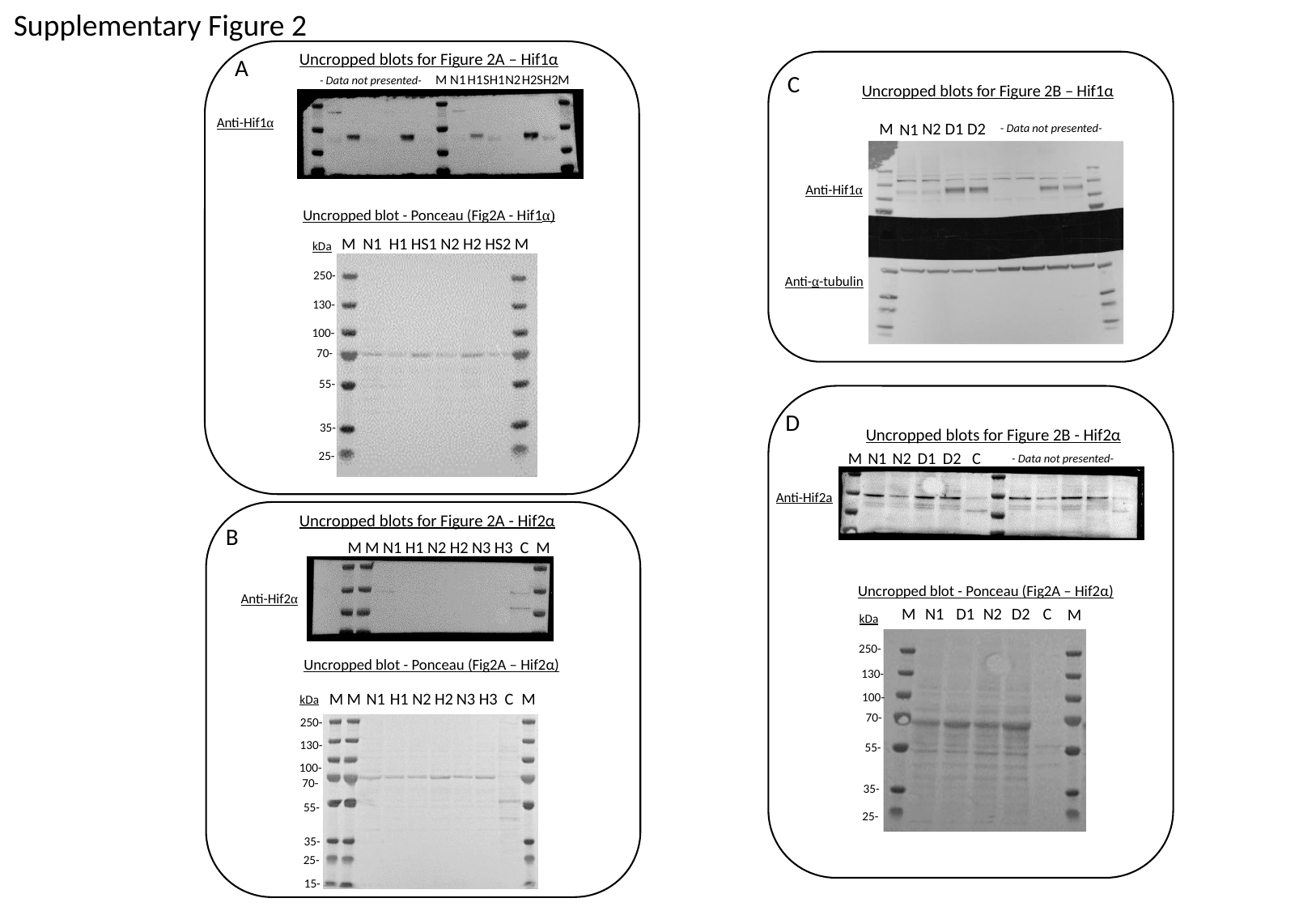

Supplementary Figure 2
Uncropped blots for Figure 2A – Hif1α
Anti-Hif1α
A
C
SH2
H1
N1
N2
SH1
M
H2
M
- Data not presented-
Uncropped blots for Figure 2B – Hif1α
D2
N2
D1
N1
M
- Data not presented-
Anti-Hif1α
Uncropped blot - Ponceau (Fig2A - Hif1α)
M N1 H1 HS1 N2 H2 HS2 M
kDa
250-
130-
100-
70-
55-
35-
25-
Anti-α-tubulin
D
Uncropped blots for Figure 2B - Hif2α
D2 C
D1
N2
N1
M
- Data not presented-
Anti-Hif2a
Uncropped blots for Figure 2A - Hif2α
B
M M N1 H1 N2 H2 N3 H3 C M
Uncropped blot - Ponceau (Fig2A – Hif2α)
Anti-Hif2α
C
N1
D1
M
N2
D2
M
kDa
250-
130-
100-
70-
55-
35-
25-
Uncropped blot - Ponceau (Fig2A – Hif2α)
M
M
N3
H2
M
C
N1
H1
N2
H3
kDa
250-
130-
100-
70-
55-
35-
25-
15-

## Slide 3
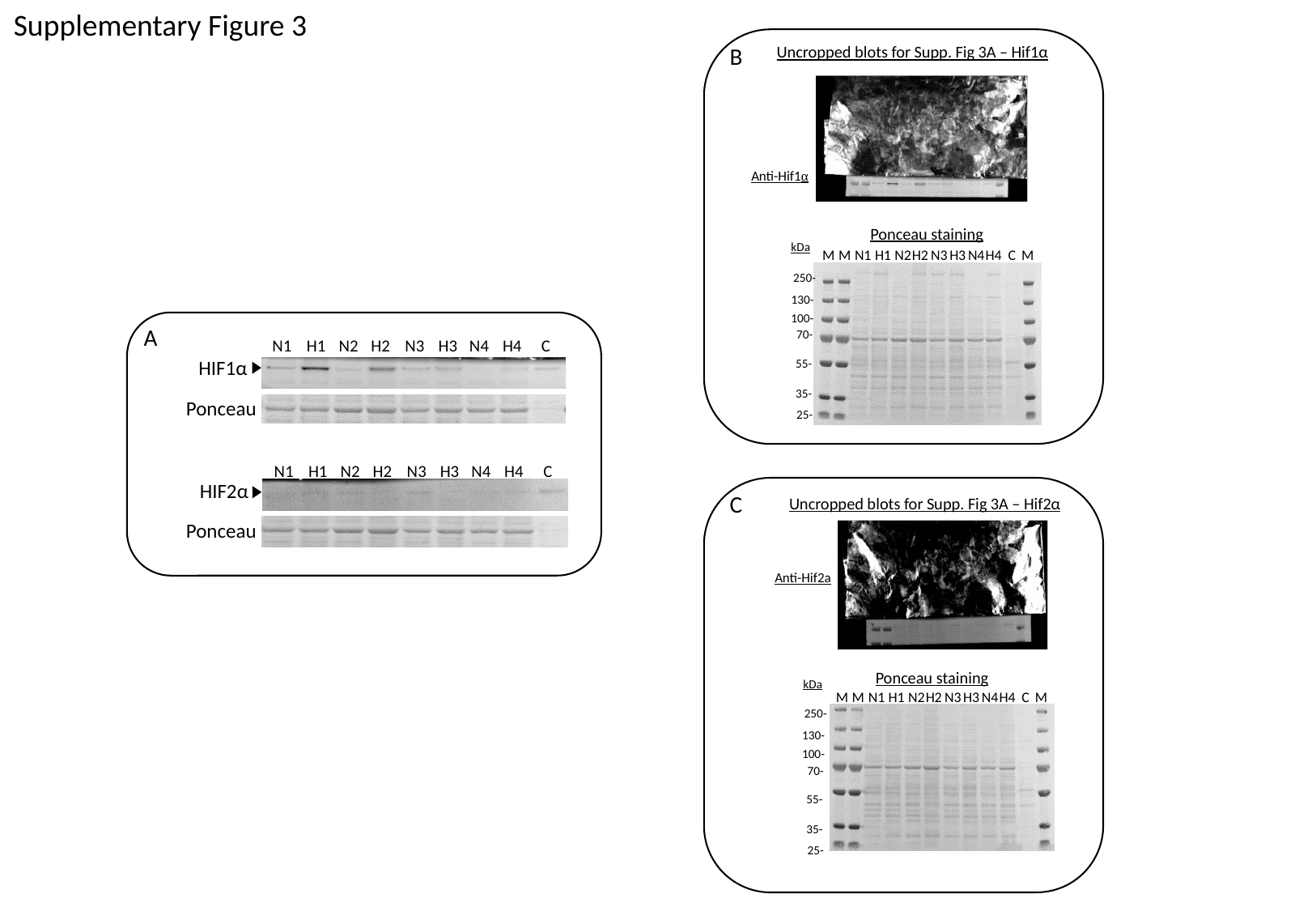

Supplementary Figure 3
B
Uncropped blots for Supp. Fig 3A – Hif1α
Anti-Hif1α
Ponceau staining
kDa
250-
130-
100-
70-
55-
35-
25-
M
M
N1
H1
N2
H2
N3
H3
N4
H4
C
M
A
C
N1
H4
N4
H3
N3
N2
H2
H1
HIF1α
Ponceau
C
N1
H4
N4
H3
N3
N2
H2
H1
HIF2α
C
Uncropped blots for Supp. Fig 3A – Hif2α
Ponceau
Anti-Hif2a
Ponceau staining
kDa
250-
130-
100-
70-
55-
35-
25-
M
M
N1
H1
N2
H2
N3
H3
N4
H4
C
M

## Slide 4
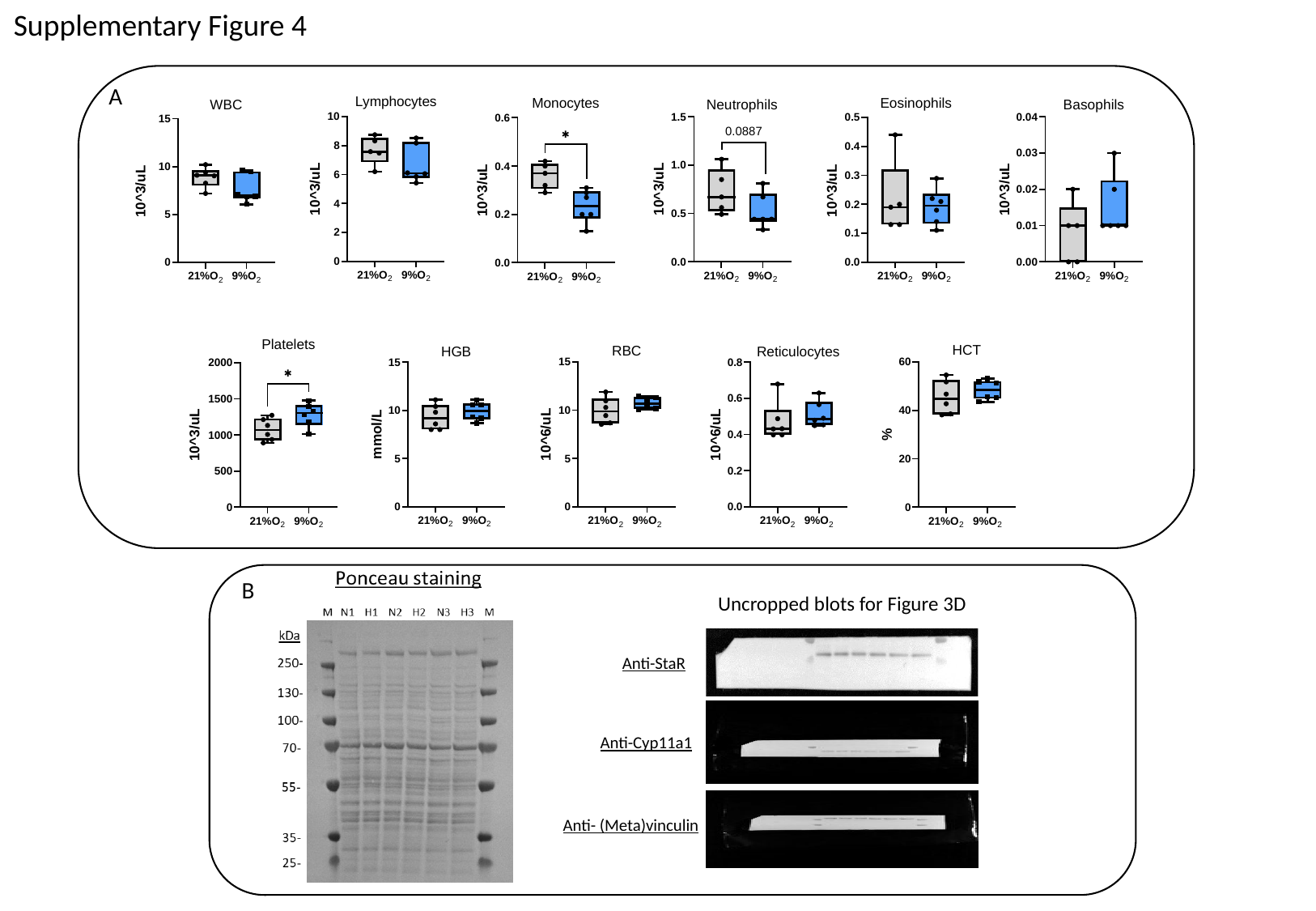

Supplementary Figure 4
A
B
Uncropped blots for Figure 3D
Anti-StaR
Anti-Cyp11a1
Anti- (Meta)vinculin

## Slide 5
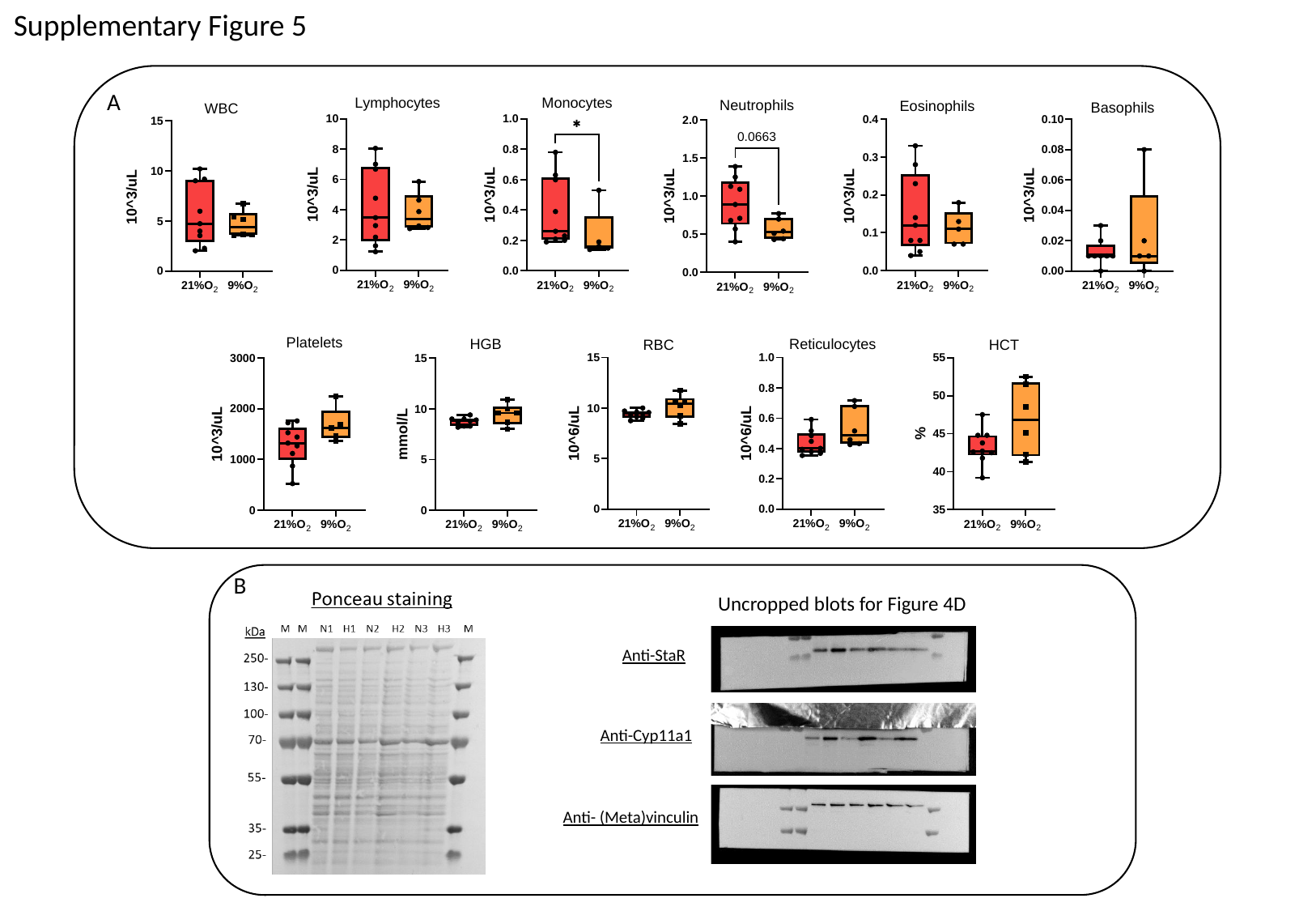

Supplementary Figure 5
A
B
Uncropped blots for Figure 4D
Anti-StaR
Anti-Cyp11a1
Anti- (Meta)vinculin
